# Prevalence of human papillomavirus antibodies in young female subjects in England

**DOI:** 10.1038/sj.bjc.6603955

**Published:** 2007-08-28

**Authors:** M Jit, A Vyse, R Borrow, R Pebody, K Soldan, E Miller

**Affiliations:** 1 Immunisation Department, Centre for Infections, Health Protection Agency, 61 Colindale Avenue, London NW9 5HT, UK; 2 Modelling and Economics Unit, Centre for Infections, Health Protection Agency, 61 Colindale Avenue, London NW9 5HT, UK; 3 Vaccine Evaluation Unit, Health Protection Agency North West Laboratory, PO Box 209, Clinical Sciences Building, Manchester Royal Infirmary, Manchester M13 9WZ, UK; 4 HIV & STI Department, Centre for Infections, Health Protection Agency, 61 Colindale Avenue, London NW9 5HT, UK

**Keywords:** England, female, papillomavirus, human, seroepidemiologic studies

## Abstract

Sera from 1483 female subjects in England aged 10–29 years were tested. The age-standardised seroprevalence was 10.7% (95% confidence intervals 9.0–12.3) for human papillomavirus (HPV) 6, 2.7% (1.8–3.6) for HPV 11, 11.9% (10.2–13.6) for HPV 16, 4.7% (3.5–5.8) for HPV 18, and 20.7% (18.6–22.7) for any of the four types.

Infection with human papillomavirus (HPV) types identified as ‘high-risk’ is a pre-requisite for developing cervical cancer (Munoz *et al*, 2003). HPV types 16 and 18 are associated with 70% of cervical cancers worldwide (Munoz *et al*, 2003), while HPV types 6 and 11, although not ‘high-risk’, are associated with over 90% of cases of anogenital warts (von Krogh *et al*, 2001). Two prophylactic vaccines against HPV (a bivalent vaccine against types 16 and 18, and a quadrivalent vaccine that also includes types 6 and 11) have been shown in clinical trials to reduce persistent HPV infection and associated disease by over 90% in up to 5 years of follow-up (Harper *et al*, 2004; Villa *et al*, 2006).

Epidemiological knowledge of HPV infection in the UK relies heavily on prevalence studies of HPV DNA in the cervical epithelium of women undergoing cervical sampling (Woodman *et al*, 2001; Kitchener *et al*, 2006) and usually relates to female subjects known to be sexually active. These studies indicate the prevalence of current infection, as most HPV infections are transient and become DNA negative within 2 years (Moscicki *et al*, 2006). In individuals who mount a detectable humoral immune response, HPV type-specific serum antibodies are an indicator of past exposure. Testing of blood samples also offers the opportunity to survey different populations.

Enzyme-linked immunosorbent assays (ELISAs) utilising virus-like particles have been used successfully for seroprevalence studies in several countries including the USA (Stone *et al*, 2002) and Sweden (af Geijersstam *et al*, 1999). We report on the first population-based study of HPV 6, 11, 16 and 18 seroprevalence in England, in 10- to 29-year-old female subjects – the likely target age range for vaccination, but an age range in which little is known about infection rates.

## MATERIALS AND METHODS

Serum specimens were obtained from the Health Protection Agency Sero-Epidemiology Unit collection, consisting of unlinked residual sera submitted to laboratories in England for routine microbiological or biochemical investigations. Sera from immuno-compromised individuals and repeat sera from the same individuals were excluded (Osborne *et al*, 2000).

Sera were selected from 1483 women aged 10–29 years, chosen as most important for informing the design of HPV vaccination programmes in the UK. Sera came from 11 laboratories in England that collected samples in 2002–2004. About 90 samples were selected for each single year of age in the range 10–19 years, and about 60 samples for each of the ages 20–29 years. Samples were tested for specific neutralising antibodies to HPV 6, 11, 16 and 18 by Merck and Co Inc., using a multiplexed competitive Luminex® assay with antibody levels reported in milli-Merck units per millilitre (mMu ml^−1^) as previously described (Opalka *et al*, 2003). Titres were calibrated to ensure comparability with other published work using the same assay (calibration factors provided by Mark Esser, personal communication). Sera were assumed to be seropositive at the cutoffs determined in previous work with this assay (Dias *et al*, 2005): 20, 16, 24 and 20 mMU ml^−1^ for HPV 6, 11, 16 and 18, respectively.

To calculate the overall seroprevalence, age-specific proportions were standardised to female population figures from the Office of National Statistics for England in 2004. Logistic regression models were used to investigate the risk of seropositivity for each HPV type, by age, source laboratory location (North or South of England) and positivity for other HPV types.

## RESULTS

**Figure 1:**
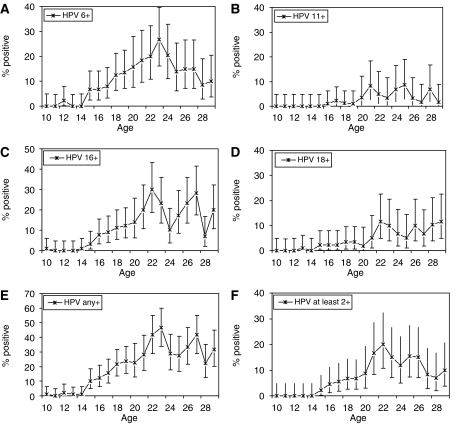
Seroprevalence of antibodies in female subjects aged 10–29 years using single year age groups for (**A**) HPV 6, (**B**) HPV 11, (**C**) HPV 16, (**D**) HPV 18, (**E**) any assayed HPV type and (**F**) at least two assayed HPV types. Error bars show exact 95% confidence intervals.

Figure 1 shows the seroprevalence of each HPV type by single year of age in our sample. The age-standardised seroprevalence in women aged 10–29 years was 10.7% (95% CI 9.0–12.3) for HPV 6, 2.7% (1.8–3.6) for HPV 11, 11.9% (10.2–13.6) for HPV 16, 4.7% (3.5–5.8) for HPV 18 and 20.7% (18.6–22.7) for any of the four assayed types. Also, 7.7% (6.3–9.1) were seropositive for at least two assayed types: 1.5% (0.9–2.2) for both HPV 6 and 11, and 2.2% (1.4–3.0) for both HPV 16 and 18. Increasing age was significantly associated with seropositivity for all HPV types (*P*<0.01). Being seropositive for one type was significantly associated with being seropositive for another (*P*<0.05), except for the case of HPV 18. HPV 18 seropositivity was only significantly associated with HPV 16 seropositivity (*P*<0.01) and not seropositivity for HPV 6 or 11. There was no consistent, significant risk of HPV seropositivity associated with sample origin from the North or South of England.

## DISCUSSION

Based on our sample, less than 5% of girls under the age of 14 years were seropositive for any HPV type. From age 14 years onwards, the seroprevalence increased sharply until the early 20s, and then stabilised or declined. The proportion seropositive varied by type, being highest for HPV 16 and lowest for HPV 11. Seropositivity for each of types 6, 11 and 18 was significantly associated with seropositivity for type 16, which is responsible for the majority of cervical cancers.

Quantitative titres from the study are not comparable to those from studies which use different assays, because currently no international HPV standard reference serum exists, and different laboratories therefore use ‘in-house’ standard sera making direct comparisons nonviable (Ferguson *et al*, 2006). However, the intended use of the data is to determine the prevalence of past HPV infection and not to analyse the dynamics of postinfection antibody levels quantitatively. In this respect, our results are broadly similar to seroprevalence data in other populations. In the United States, 6.8 and 24.7% of 12–19 and 20–29 years old female subjects, respectively, were found by ELISA to have HPV 16 antibodies (Stone *et al*, 2002). Our results are also consistent with the onset of sexual activity in the UK, as described by a sexual behaviour study in 2000 which found that 50% of women reported sexual debut by age 17 years (Wellings *et al*, 2001). The results for HPV 16 and 18 in women of ages eligible for cervical screening consistently exceeded the proportion of women found to be HPV DNA positive in a recent study of residual cervical smear samples (Kitchener *et al*, 2006), as expected for a persistent marker of previous infection.

There are a number of issues to consider before using these data to estimate the incidence of HPV infection. Firstly, seroprevalence is likely to underestimate the proportion of women who have had an HPV infection, since other studies have suggested that only 65–90% of HPV DNA-positive female subjects seroconvert, with differences in estimates depending on factors including the testing systems used, how long HPV DNA persists and whether or not there is progression to disease (Dillner, 1999; Carter *et al*, 2000). Seroconversion may coincide with DNA detection, or may follow by some months; for example, one study found a delay in seroconversion of 6–12 months after HPV 16 infection, with type-specific variation seen in the time to seroconversion (Carter *et al*, 2000).

The population tested in the survey is not randomly selected but consists of individuals accessing health care in England and having blood samples taken for diagnostic or screening investigations. Previous studies using the same collection have, however, found a consistency between seroprevalence for other vaccine preventable infections and population vaccine coverage data (Osborne *et al*, 2000). This suggests that the sample source is broadly representative of the general population, at least for relatively common infections. An Australian study in school-aged children has shown that samples from diagnostic laboratories can give estimates of immunity to vaccine preventable diseases that are comparable to random cluster surveys in the general population (Kelly *et al*, 2002).

The apparent decline in seroprevalence observed around the age of 25 years suggests that seroprevalence is not a straightforward marker of all past HPV infection. Such a marker would be expected to continue to rise in older women, since DNA studies show evidence of incident HPV infections in women of all ages, albeit at a lower rate in older women (Kitchener *et al*, 2006). There may be several possible explanations for this. Firstly, the female subjects in this survey were from different birth cohorts, so changes in sexual behaviour in the 1970s and 1980s would be reflected in the results, as has been observed in a similar study in Sweden (af Geijersstam *et al*, 1999). The population tested in the survey may differ by age in terms of how well they represent their age cohort with respect to HPV infection. Finally, antibody levels are likely to wane over time, as has been suggested in other studies (Carter *et al*, 2000). The use of seroprevalence data such as these to estimate age-specific infection rates therefore needs to be investigated further, for example by comparing force of infection estimates under different assumptions about waning antibody levels to prevalence data from DNA studies. Further work involving testing of similar samples from older females (aged 30–49 years) should also inform the pattern of HPV acquisition throughout life.

Despite these complications, these data offer an additional viewpoint on the epidemiology of HPV in England, and a rare view of the age-dependent risk of HPV infection in female subjects who are not selected for their known sexual activity and who are below the age eligible for routine cervical screening and in the age range likely to be targeted with HPV vaccination. They suggest that there is already a substantial risk of HPV infection in girls in England by the age of 14 years, which has implications for the age at which vaccination should be delivered. These data and further analyses should help to determine the most effective strategies for routine and catch-up HPV vaccination.

## References

[bib1] af Geijersstam V, Eklund C, Wang Z, Sapp M, Schiller JT, Dillner J, Dillner L (1999) A survey of seroprevalence of human papillomavirus types 16, 18 and 33 among children. Int J Cancer 80: 489–493 9935145 10.1002/(sici)1097-0215(19990209)80:4<489::aid-ijc1>3.0.co;2-9

[bib2] Carter JJ, Koutsky LA, Hughes JP, Lee SK, Kuypers J, Kiviat N, Galloway DA (2000) Comparison of human papillomavirus types 16, 18, and 6 capsid antibody responses following incident infection. J Infect Dis 181: 1911–1919 10837170 10.1086/315498

[bib3] Dias D, Van Doren J, Schlottmann S, Kelly S, Puchalski D, Ruiz W, Boerckel P, Kessler J, Antonello JM, Green T, Brown M, Smith J, Chirmule N, Barr E, Jansen KU, Esser MT (2005) Optimization and validation of a multiplexed luminex assay to quantify antibodies to neutralizing epitopes on human papillomaviruses 6, 11, 16, and 18. Clin Diagn Lab Immunol 12: 959–969 16085914 10.1128/CDLI.12.8.959-969.2005 PMC1182182

[bib4] Dillner J (1999) The serological response to papillomaviruses. Semin Cancer Biol 9: 423–430 10712889 10.1006/scbi.1999.0146

[bib5] Ferguson M, Heath A, Johnes S, Pagliusi S, Dillner J (2006) Results of the first WHO international collaborative study on the standardization of the detection of antibodies to human papillomaviruses. Int J Cancer 118(6): 1508–1514 16184553 10.1002/ijc.21515

[bib6] Harper DM, Franco EL, Wheeler C, Ferris DG, Jenkins D, Schuind A, Zahaf T, Innis B, Naud P, De Carvalho NS, Roteli-Martins CM, Teixeira J, Blatter MM, Korn AP, Quint W, Dubin G (2004) Efficacy of a bivalent L1 virus-like particle vaccine in prevention of infection with human papillomavirus types 16 and 18 in young women: a randomised controlled trial. Lancet 364: 1757–1765 15541448 10.1016/S0140-6736(04)17398-4

[bib7] Kelly H, Riddell MA, Gidding HF, Nolan T, Gilbert GL (2002) A random cluster survey and a convenience sample give comparable estimates of immunity to vaccine preventable diseases in children of school age in Victoria, Australia. Vaccine 20: 3130–3136 12163264 10.1016/s0264-410x(02)00255-4

[bib8] Kitchener HC, Almonte M, Wheeler P, Desai M, Gilham C, Bailey A, Sargent A, Peto J (2006) HPV testing in routine cervical screening: cross sectional data from the ARTISTIC trial. Br J Cancer 95: 56–61 16773068 10.1038/sj.bjc.6603210 PMC2360499

[bib9] Moscicki AB, Schiffman M, Kjaer S, Villa LL (2006) Chapter 5: updating the natural history of HPV and anogenital cancer. Vaccine 24(Suppl 3): S42–S51 10.1016/j.vaccine.2006.06.018 16950017

[bib10] Munoz N, Bosch FX, de Sanjose S, Herrero R, Castellsague X, Shah KV, Snijders PJ, Meijer CJ (2003) Epidemiologic classification of human papillomavirus types associated with cervical cancer. N Engl J Med 348: 518–527 12571259 10.1056/NEJMoa021641

[bib11] Opalka D, Lachman CE, MacMullen SA, Jansen KU, Smith JF, Chirmule N, Esser MT (2003) Simultaneous quantitation of antibodies to neutralizing epitopes on virus-like particles for human papillomavirus types 6, 11, 16, and 18 by a multiplexed luminex assay. Clin Diagn Lab Immunol 10: 108–115 12522048 10.1128/CDLI.10.1.108-115.2003 PMC145272

[bib12] Osborne K, Gay N, Hesketh L, Morgan-Capner P, Miller E (2000) Ten years of serological surveillance in England and Wales: methods, results, implications and action. Int J Epidemiol 29: 362–368 10817137 10.1093/ije/29.2.362

[bib13] Stone KM, Karem KL, Sternberg MR, McQuillan GM, Poon AD, Unger ER, Reeves WC (2002) Seroprevalence of human papillomavirus type 16 infection in the United States. J Infect Dis 186: 1396–1402 12404154 10.1086/344354

[bib14] Villa LL, Costa RL, Petta CA, Andrade RP, Paavonen J, Iversen OE, Olsson SE, Hoye J, Steinwall M, Riis-Johannessen G, Andersson-Ellstrom A, Elfgren K, Krogh G, Lehtinen M, Malm C, Tamms GM, Giacoletti K, Lupinacci L, Railkar R, Taddeo FJ, Bryan J, Esser MT, Sings HL, Saah AJ, Barr E (2006) High sustained efficacy of a prophylactic quadrivalent human papillomavirus types 6/11/16/18 L1 virus-like particle vaccine through 5 years of follow-up. Br J Cancer 95: 1459–1466 17117182 10.1038/sj.bjc.6603469 PMC2360730

[bib15] von Krogh G, Lacey CJ, Gross G, Barrasso R, Schneider A (2001) European guideline for the management of anogenital warts. Int J STD AIDS 12(Suppl 3): 40–47 11589796 10.1258/0956462011924100

[bib16] Wellings K, Nanchahal K, Macdowall W, McManus S, Erens B, Mercer CH, Johnson AM, Copas AJ, Korovessis C, Fenton KA, Field J (2001) Sexual behaviour in Britain: early heterosexual experience. Lancet 358: 1843–1850 11741623 10.1016/S0140-6736(01)06885-4

[bib17] Woodman CB, Collins S, Winter H, Bailey A, Ellis J, Prior P, Yates M, Rollason TP, Young LS (2001) Natural history of cervical human papillomavirus infection in young women: a longitudinal cohort study. Lancet 357: 1831–1836 11410191 10.1016/S0140-6736(00)04956-4

