# Prevalence of human papillomavirus antibodies in young female subjects in England

**DOI:** 10.1038/sj.bjc.6604403

**Published:** 2008-05-05

**Authors:** M Jit, A Vyse, R Borrow, R Pebody, K Soldan, E Miller

**Correction to**: *British Journal of Cancer* (2007) **97**, 989–991. doi: 10.1038/sj.bjc.6603955

There was an error in the second paragraph, last sentence, of the Materials and Methods section of this paper, published in Volume 97, Number 7 (October 2007).

The corrected sentence should read:

Sera were assumed to be seropositive at the cutoffs determined in previous work with this assay (Dias *et al*., 2005): 20, 16, 20 and 24 mMUml for HPV 6, 11, 16 and 18, respectively.

